# Blocked expression of key genes of the angiogenic pathway in JSRV-induced pulmonary adenocarcinomas

**DOI:** 10.1186/s13567-017-0480-z

**Published:** 2017-11-14

**Authors:** Maryline Gomes, Fabienne Archer, Nicolas Girard, Barbara Gineys, Christine Dolmazon, Alexandra Bobet Erny, Jean-François Mornex, Caroline Leroux

**Affiliations:** 1 IVPC UMR754, INRA, Univ Lyon, Université Claude Bernard Lyon 1, EPHE, Lyon, France; 2 Department of Respiratory Diseases, Hospices Civils de Lyon, Louis Pradel Hospital, Lyon, France

## Abstract

JSRV (Jaagsiekte Sheep Retrovirus) is a retrovirus inducing a transmissible lung adenocarcinoma in sheep and goats with predominantly lepidic and papillary lesions. This naturally occurring lung cancer in large animals shares many features with human pneumonic-type lung adenocarcinomas with predominant lepidic growth. The metastatic spread is rare in both human and animal cancers. This unique feature prompted us to decipher the angiogenesis pathway in these cancers. We focused on the levels of mRNA and proteins of genes implicated in the extension of JSRV-induced lung adenocarcinomas by studying their expression in lung cancers (*n* = 10) and normal lungs (*n* = 10) and in primary epithelial alveolar type II cells derived from cancers (*n* = 10) or normal lungs (*n* = 6). In parallel, we evaluated the levels of expression of key genes in lung tissues collected from lepidic (*n* = 13) or papillary (*n* = 5) human adenocarcinomas and, when available, adjacent normal lungs (*n* = 11). We measured the expression of the same key genes implicated in angiogenesis, lymphangiogenesis and degradation of the extracellular matrix. In ovine adenocarcinomas, *VEGFR2* and *VEGFD* mRNA were downregulated in cancers; *MMP9*, *TIMP1* and *FGFR2* mRNA were overexpressed as compared to normal lungs. Importantly, VEGFA and VEGFR2 proteins were not expressed in JSRV-induced cancers. In human lepidic adenocarcinomas, *VEGFA* and *VEGFR2* mRNA were weakly expressed and no VEGFR2 protein was detectable. Downregulation of key angiogenic players may contribute to the control of extra thoracic invasion of cancer cells in human and ovine pneumonic-type adenocarcinoma with predominant lepidic growth.

**Electronic supplementary material:**

The online version of this article (10.1186/s13567-017-0480-z) contains supplementary material, which is available to authorized users.

## Introduction

Cancers develop as the result of deregulation of multiple cell processes such as resistance to cell death, independence from growth suppressors and inflammation [1]. Angiogenesis and lymphangiogenesis mechanisms are involved in the spreading of cancer cells. Angiogenesis is the neo-formation of blood vessels from pre-existing ones and is a highly-regulated mechanism [2]. This cell mechanism is crucial for the extension of cancers and is a major target for drug development [3]. When angiogenic pathways are activated, cancer cells communicate with endothelial cells through pro-angiogenic factors that activate the proliferation of endothelial cells. Activation of angiogenesis is controlled by the release of a wide array of intercellular mediators by cancer cells. The main angiogenesis signaling pathway is triggered by VEGF (Vascular Endothelial Growth Factor) and ligation to the VEGF receptors. While VEGFA, B, C and D are secreted by cancer cells, their receptors VEGFR1, R2 and R3 are expressed at the surface of endothelial cells [4]. Ligands bind to their receptors inducing their dimerization and auto-phosphorylation; the activation of signal transduction results in endothelial cell survival and expression of genes involved in cell proliferation [4]. VEGFA was the first-identified member of the VEGF family and is expressed by various cell types including cancer cells [5]. VEGFA is the main inducer of angiogenesis and proliferation and migration of cancer cells; thereby the VEGFA/VEGFR2 complex is a key player for the activation of angiogenesis [6]. VEGFA stimulates the growth of the vascular network that supports cancer growth and metastasis and is often up-regulated in cancers [5]. On the contrary, lymphangiogenesis participates in dissemination of cancer cells by its activation of the growth of lymphatic vessels through the interaction of VEGFC or VEGFD ligands with VEGFR3 receptors expressed at the surface of lymphatic endothelial cells. VEGFC and VEGFD can also activate angiogenesis through their interaction with VEGFR2 [7]. NRP1 and NRP2 (Neuropilin 1 and 2) are VEGFR co-receptors that potentiate VEGF fixation on VEGFRs [8]. Finally, the degradation of the extracellular matrix is essential for cancer growth and progression through proteolytic activities that facilitate the growth or spread of new blood and lymphatic vessels into the primary cancer [2]. The activity of extracellular matrix proteases is under tight control in normal conditions, but cancer cells are prone to activate the proteolytic activities of PLAU (Plasminogen Activator Urokinase), MMP (Matrix Metallopeptidase) 2 and 9, involved in extracellular matrix degradation during cancer invasion process and angiogenesis [9, 10]. SERPINE1 (Serpin family E member 1) and TIMP1 (Tissue Inhibitor of MetalloProteinase 1) are inhibitors of PLAU and MMP9 respectively. Other pathways are implicated in the regulation of angiogenesis such as the FGF pathway controlled by FGF (Fibroblast Growth Factor) ligands and their receptors [11].

Ovine pulmonary adenocarcinoma is a virally induced lung cancer affecting sheep and goats. The JSRV (Jaagsiekte Sheep RetroVirus) β-retrovirus responsible for the disease is transmitted by aerosols, milk, colostrum and in utero [12–14]. JSRV infects and transforms cells through its envelope, carrying the oncogenic properties. The retrovirus solely transforms epithelial cells from the distal lung namely AECIIs (Alveolar Epithelial type II Cells) in the alveoli and Club cells in the bronchiole. Ovine pulmonary adenocarcinoma shares striking similarities with human pneumonic-type lung adenocarcinoma (PTLA) with predominant lepidic growth [15–17]. This growth pattern refers to the lining of cancer cells along the alveolar septa without disorganization of the alveolar architecture and with no evidence of stromal, vascular or pleural invasion. Lepidic adenocarcinoma affects more non-smokers, women and young subjects than other lung adenocarcinomas [18]. These cancers are slow-growing and as with ovine pulmonary adenocarcinoma, metastatic spread is rare. This unique feature prompted us to decipher the angiogenesis pathway in JSRV-induced pulmonary adenocarcinomas and human lepidic adenocarcinomas especially when considering that most lung cancers in humans are highly invasive [19] and that the activation of angiogenesis is associated with a poor prognosis [20].

Our data showed that the expression of key genes implicated in angiogenesis were dramatically downregulated in JSRV-induced lung cancers. Protein expression of VEGFA and of its receptor VEGFR2 was almost abolished in cancers while *VEGFB* and *VEGFD* mRNA expression were significantly reduced. At the same time, expression of *TIMP1*, inhibiting the metalloproteinases, was increased. Similarly, VEGFA was weakly expressed and VEGFR2 was not expressed in human lepidic adenocarcinomas. Moreover, mRNA expression *VEGFD*, *FGF2 MMP2*, *NRP1* and *NRP2* was decreased in lepidic adenocarcinomas when compared to the normal lungs. Downregulation of key angiogenic players may contribute to the control of extra thoracic invasion of cancer cells in lepidic-predominant adenocarcinomas both in sheep and humans.

## Materials and methods

### Biological materials

Ovine pulmonary adenocarcinoma specimens were collected *post mortem* from twenty-five spontaneously infected sheep with clinical evidence of lung cancer obtained from flocks in southern France and from twenty-one sheep without clinical signs of cancer obtained from the Corbas slaughterhouse, nearby Lyon (France) (Table 1). Formal authorization for accessing the facilities was obtained and access was granted under the supervision of a veterinarian. None of the animals used in this study were engaged in an experimental protocol. Clinical status was confirmed by the presence of gross lesions and by the detection of JSRV genome using semi-nested PCR with primers located in *env* and LTR regions, generated from sequences of field isolates as previously described [21]. All cancer samples were positive for JSRV proviral DNA and were pathologically described as lepidic predominant with papillary subtypes. When possible, primary alveolar epithelial type II cells (AECII) were derived from the tissues. Seventeen primary AECII cultures were obtained from lung cancers and sixteen from normal lungs (Table 1). The lung-derived ovine AECII have been phenotypically characterized for the expression of pro-SP-C and cytokeratin and cultured as previously described [22]. We studied the mRNA and protein expression in lungs and lung-derived AECII from the same animals when possible; unfortunately, due to the limited number of primary cells directly derived from cancer or normal lungs, this was not possible in all cases. When necessary, we used AECII cultures from other animals from our biobank.

**Table 1:** **Lung samples from sheep with or without pulmonary adenocarcinomas**

With lesion of pulmonary adenocarcinoma					Without lesion				
Studied samples					Studied samples				
#	Sex	Age (months)	Lung	Derived AECIIs	#	Sex	Age (months)	Lung	Derived AECIIs
1167	F	60	No	Yes	1010	–	< 6	Yes	Yes
1171	F	–	No	Yes	1011	–	< 6	Yes	Yes
1220	F	–	No	Yes	1045	–	ND	No	Yes
1221	F	–	No	Yes	1124	–	< 6	No	Yes
1295	F	72	No	Yes	1169	–	< 1	No	Yes
1296	F	24	No	Yes	1223	–	< 6	Yes	Yes
1297	F	15	No	Yes	1224	–	< 6	Yes	Yes
1298	F	15	No	Yes	1472	–	< 6	No	Yes
1468	F	–	No	Yes	1486	–	< 1	No	Yes
1481	–	–	No	Yes	1487	–	< 1	Yes	Yes
1482	F	–	No	Yes	1507	–	< 1	No	Yes
2055	–	–	Yes	No	1508	–	< 1	No	Yes
2334	F	–	Yes	No	1823	–	< 6	Yes	No
2335	–	–	Yes	No	1824	–	< 6	Yes	No
2339	M	–	Yes	No	1825	–	< 6	Yes	No
2369	F	–	Yes	No	2340	–	< 6	Yes	No
2371	F	–	Yes	No	2355	–	< 6	Yes	No
2433	–	–	Yes	Yes	2645	–	< 6	No	Yes
2374	–	–	No	Yes	2649	–	< 6	No	Yes
2434	F	18	Yes	No	2650	–	< 6	No	Yes
2436	–	–	Yes	Yes	2652	–	< 6	No	Yes
2479	F	–	Yes	No					
2528	M	–	No	Yes					
2529	M	–	No	Yes					
2585	M	12	No	Yes					

Lungs were obtained from slaughterhouse or flocks with endemic cases of cancers. For these reasons, the data collected were incomplete.

“–”: missing data.

Human lung specimens from 18 patients (13 lepidic and five papillary adenocarcinomas) and their associated data were obtained from Cardiobiotec Biobank (CRB-HCL Hospices Civils de Lyon BB-0033-00046), a center for biological resources authorized by the French Ministry of Social Affairs and Health. All samples were collected and used in accordance with the ethical rules of the Biobank and in agreement with French legislation. All patients signed a written informed consent. When available in the Biobank, adjacent normal lung was collected and analyzed (Table 2).

**Table 2:** **Lung samples from human lung adenocarcinomas**

#	Sex	Age (years)	TNM	Mutation status						Mucine status	Analyzed tissues	
				EGFR	KRAS	BRAF	ERBB2	PIK3CA	ALK		Tumoral	Normal
Lepidic												
923	Female	50	T1bN0M0	ND	ND	ND	ND	ND	ND	Nonmucinous	Yes	No
974	Male	58	T4N0M0	ND	ND	ND	ND	ND	ND	Unspecified	Yes	No
975	Female	79	a	ND	ND	ND	ND	ND	ND	Mucinous	Yes	No
979	Female	76	ND	ND	ND	ND	ND	ND	ND	Unspecified	Yes	No
1030	Female	53	T1bN0M0	ND	ND	ND	ND	ND	ND	Unspecified	Yes	No
1042	Male	40	T1bN0M0	ND	ND	ND	ND	ND	ND	Unspecified	Yes	No
2363	Male	53	T2bN0M0	ND	ND	ND	ND	ND	ND	Unspecified	Yes	No
2532	Female	66	T1aN0M0	–	+	–	–	–	–	Nonmucinous	Yes	Yes
2533	Male	78	T3N0M0	–	**–**	+	–	–	–	Mucinous	Yes	Yes
2534	Male	46	T1bN0M0	–	+	–	–	–	–	Nonmucinous	Yes	Yes
2535	Female	76	T1aN0M0	–	–	–	–	–	–	Nonmucinous	Yes	Yes
2537	Male	62	T2aN0M0	–	ND	+	–	–	–	Nonmucinous	Yes	Yes
2538	Male	54	T2bN0M0	–	+	–	–	–	–	Mixed	Yes	Yes
Papillary												
2536	Female	54	T3N2M0	–	ND	+	–	–	–	Mucinous	Yes	Yes
2539	Male	61	T3N0M0	–	ND	ND	ND	ND	ND	ND	Yes	Yes
2540	Male	61	T3N1M0	–	ND	ND	ND	ND	ND	ND	No	Yes
2541	Male	63	T1N0M0	–	–	–	–	–	–	ND	Yes	Yes
2542	Female	51	T1N0M0	–	–	+	–	–	–	ND	Yes	Yes
2543	Female	83	T2bN2M0	–	–	+	–	–	ND	ND	Yes	No

ND: not determined (or not known), TNM: tumor node metastasis staging.

^a^Lesions from patient 975 were described as predominantly lepidic with a contingent of acinar lesions; the TNM was not determined.

### Expression of genes involved in angiogenesis, lymphangiogenesis and extracellular-matrix degradation

Total RNA from 20 ovine and 29 human lung tissues were extracted using the “RNeasy Plus Universal Mini” kit (Qiagen) and total RNA from ovine primary AECII cultures (*n* = 16) were extracted using the “PureLink RNA mini” kit and treated with the “Turbo DNA free” kit (Ambion) as recommended. One microgram of total RNA was reverse transcribed using the “iScript cDNA synthesis” kit (Biorad). The cDNAs were amplified using the “KAPA SYBR® FAST Universal qPCR kit” (Clinisciences) in triplicate using a MiniOpticon thermocycler (Biorad) with an initial denaturation step at 95 °C for 3 min, followed by 40 cycles of 5 s at 95 °C and 20 s at 60 °C. The melting curves were obtained by a temperature increase from 60 to 95 °C with fluorescence reading every 0.5 °C.

The primers for genes coding for ligands (*VEGFA*, *VEGFB*, *VEGFC*, *VEGFD*, *FGF1*, *FGF2*), receptors (*VEGFR1*, *VEGFR2*, *VEGFR3*, *FGFR1*, *FGFR2*, *NRP1*, *NRP2*), peptidases and peptidase inhibitors (*MMP2*, *MMP9*, *PLAU*, *TIMP1*, *SERPINE1*) and reference genes (*PPIA*, *PSMB2*, *RPS11*, *YWHAZ*) (Additional file 1) were designed with Primer-BLAST from the available GenBank sequences with a primer spanning an exon–exon junction or with forward and reverse primers into two different exons to avoid interfering amplifications from residual DNA. The efficiency of the primers was evaluated by amplification of 10-fold dilutions of cDNA. All primer sets used in our study have a calculated efficiency of 95 to 110%. Three reference genes were selected from our set of 10 genes (not shown), described as “stably expressed” in humans or animals. Their stability in ovine and human lungs has been evaluated with the geNorm software [23]. This preliminary study (data not shown) allowed us to select *PPIA* (peptidyl isomerase A), *PSMB2* (proteasome subunit beta 2) and *YWHAZ* (tyrosine 3-monooxygenase/tryptophan 5-monooxygenase activation protein, zeta) as the most stable reference genes for mRNA quantification in sheep, and *PPIA*, *PSMB2* and *RPS11* (ribosomal protein S11) as the most stable ones for humans (Additional file 1). The normalization was performed against the three reference genes selected for their stability in sheep or humans. Normalized relative expression levels were calculated with the 2^−ΔΔCq^ method [24].

### Determination of the virus load by quantitative RT-PCR

Virus load was quantified by RT-qPCR from total RNA extracted from lungs or culture AECIIs using primers specific for exogenous JSRV, located at the end of the *env* gene for the forward primer (5′-GAGTTGAAATGCTGCATATG-3′) and in the LTR for the reverse primer (5′-GGATTCTTACACAATCACC-3′) as we previously described [21]. The virus load was measured in reference to a standard curve generated with a known number of DNA copies of the JSRV target, i.e. the cloned env-LTR amplicon carrying a 50 bp deletion. The standard curve and the total mRNA were amplified using the “KAPA SYBR® FAST Universal qPCR kit” (Clinisciences) in triplicate using a MiniOpticon thermocycler (Biorad) with an initial denaturation step at 95 °C for 3 min, followed by 40 cycles of 5 s at 95 °C and 20 s at 60 °C. The melting curves were obtained by a temperature increase from 60 °C to 95 °C with fluorescence reading every 0.5 °C. Virus load was expressed as the number of viral RNA per ng of total RNA.

### Analysis of VEGFA and VEGFR2 protein expression

Total proteins were extracted from ovine and human lung tissues and ovine primary cells. Prior to extraction, lung tissues were dissociated in T-PER lysis buffer (ThermoScientific) using the “FastPrep” device (MP Biomedicals) as recommended. After an incubation of 30 min on ice, lysates were disrupted by four sonication cycles for 20 s at 300 W (Bioblock, Fisher Scientific). Supernatants were collected by centrifugation, and their protein contents quantified using the “Quick Start TM Bradford 1X Dye Reagent” kit (Biorad) as recommended. After heat denaturation, 15–20 µg of total proteins were separated by electrophoretic migration on SDS-PAGE and transferred onto a nitrocellulose membrane. The membranes were pre-incubated with TBST-milk (25 mM Tris pH 7.6; 0.15 M NaCl; 0.05% Tween 20, 5% non-fat dry milk) for 1 h at room temperature. After three washes in TBST, membranes were incubated overnight at 4 °C with specific antibodies. The VEGFA and VEGFR2 proteins were detected with a 1/500 dilution in TBST-milk of rabbit anti-VEGFA antibodies (ab46154, Abcam) and of rabbit anti-VEGFR2 antibodies (ab39256, Abcam). After three washes with TBST, membranes were incubated for 1 h at room temperature with a 1/10 000 dilution of anti-rabbit IgG (whole molecule)-peroxidase antibodies (A0545, Sigma). Detection of β-actin (A3854, Sigma) was performed as a loading control. Immunoreactive bands were revealed with the “SuperSignal West Pico reagent” (Thermofisher). Detection of VEGFA and VEGFR2 was repeated three times and the protein expression was semi-quantified by densitometry using the image processing software UN-SCAN-IT™ (Silk Scientific Corporation).

### Statistical analysis

Statistical analyses were performed using Wilcoxon, Student or Welsh tests using R software. All tests were done with a significance threshold of 0.05.

## Results

### The VEGF pathway is downregulated in JSRV-induced adenocarcinomas

**Figure 1:**
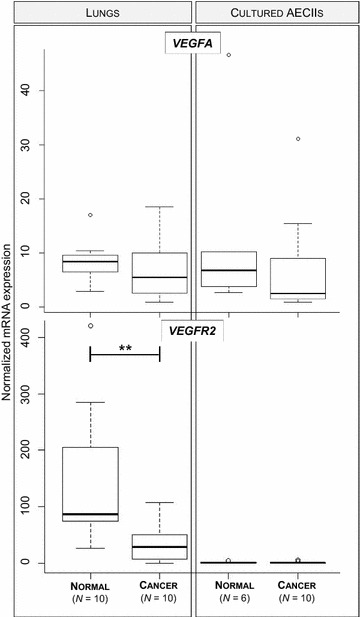
***VEGFA*** **and** ***VEGFR2*** **mRNA expression in JSRV-induced adenocarcinoma.** The mRNA expression of ovine *VEGFA* and *VEGFR2* was analyzed by RT-qPCR in lungs (10 normal lungs and 10 cancers) and lung-derived AECII cultures (6 from normal lungs and 10 from cancers). Statistical analysis was performed using the Wilcoxon test with **p < 0.005. The mRNA expression of the cellular genes was normalized with expression of the PPIA, PSMB2 and YWHAZ reference genes.

**Figure 2:**
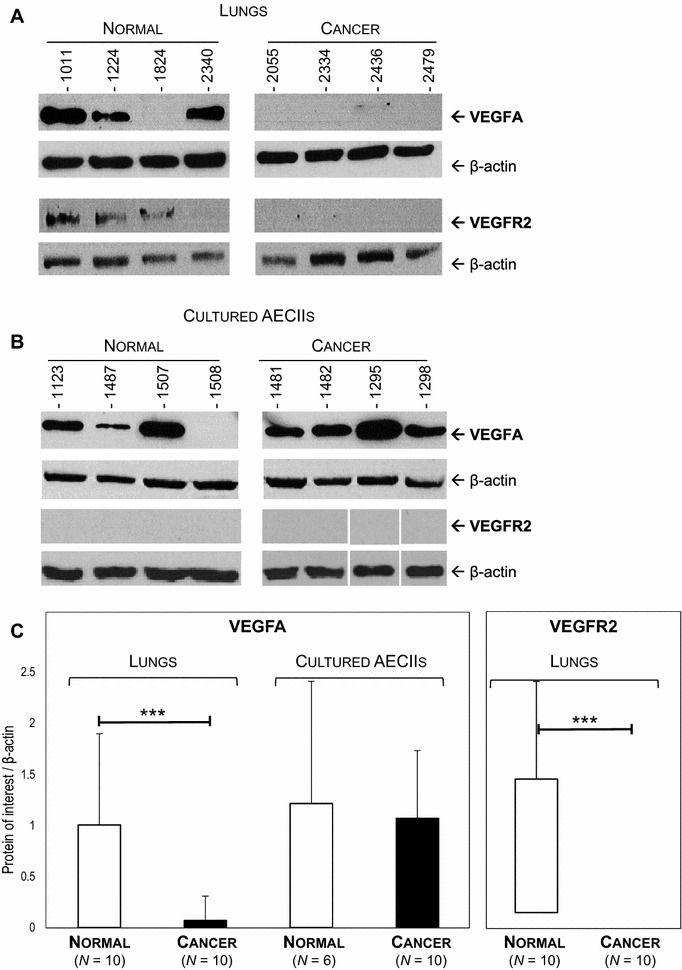
**Downregulation of VEGFA and VEGFR2 protein expression in JSRV-induced adenocarcinoma.** Levels of protein expression of VEGFA and VEGFR2 were analyzed by Western blot in lungs (**A**) and cultured AECIIs (**B**). **C** Protein expression of each sample was semi-quantified by densitometry and a ratio [protein of interest/β-actin] was calculated. Statistical analysis was performed using Student *t*-test or the Wilcoxon test with ***p < 0.0005.

We analyzed the levels of mRNA expression of *VEGFA* using RT-qPCR and its protein expression by Western blot in normal lungs, cancers, and lung-derived AECII cultures. The level of *VEGFA* mRNAs was not significantly altered in JSRV-induced cancers or the derived-AECII cultures when compared to normal samples (Figure 1). Interestingly, the expression of VEGFA protein was abolished in most cancers (90%), while the protein was expressed in 80% normal lungs (Figures 2A and C). AECII cultures derived from normal lungs or cancers both expressed VEGFA at a similar level (Figures 2B and C), suggesting that the in vitro conditions might have modified the expression of VEGFA proteins.

The mRNA expression of *VEGFR2*, the main receptor of VEGFA, was significantly reduced in cancers as compared to normal lungs and undetectable in AECII cultures derived from cancers or normal lungs (Figure 1). Correlating with the decrease of mRNA expression, VEGFR2 proteins were undetectable in cancers but present in 80% of normal lungs (Figures 2A and C). VEGFR2 protein expression was null in primary AECIIs derived from normal lungs or cancers (Figure 2B). These results highlight the loss of expression of VEGFA and of its main receptor VEGFR2 in JSRV-induced pulmonary adenocarcinomas.

**Figure 3:**
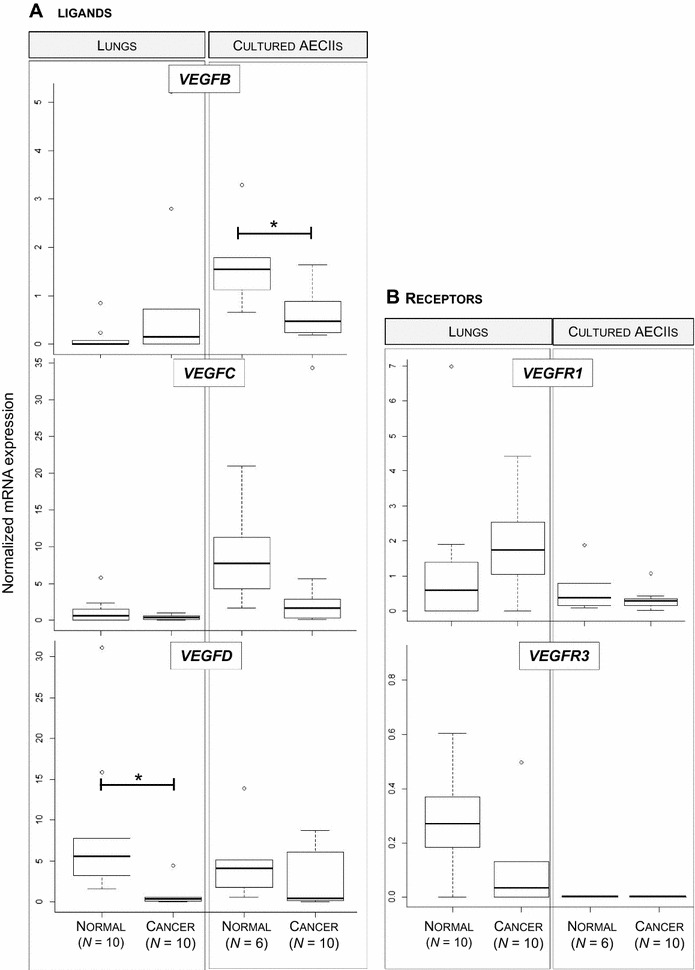
**Alteration of VEGF/VEGFR mRNA expression in JSRV- induced adenocarcinomas.** The mRNA expression of ligands *VEGFB*, *VEGFC* and *VEGFD* (**A**), and their receptors *VEGFR1* and *VEGFR3* (**B**) were analyzed by RT-qPCR in lungs and lung-derived AECII cultures. Statistical analysis was performed using Student *t*-test or the Wilcoxon test with *p < 0.05.

We measured mRNA expression of *VEGFB*, *VEGFC*, *VEGFD* ligands, of their receptors *VEGFR1*, *VEGFR*3 and of their coreceptors *NRP1* and *NRP2*. Except for the expression of *VEGFD*, which was significantly reduced in JSRV-induced adenocarcinomas, none of the other genes were differentially expressed in cancers (Figure 3). Expression of *VEGFB* and *VEGFC* was low to undetectable in most tested tissues (Figure 3A). Expression of the *VEGFR1* and *VEGFR3* receptors (Figure 3B) and *NRP1* and *NRP2* (data not shown) was not modified in JSRV-induced cancers when compared to normal lungs. Expression of *VEGFB* mRNA was significantly reduced by 2.5-fold in primary AECII derived from cancers as compared to cells derived from normal lungs and *VEGFC* expression was downregulated in cultured AECII (*p* value at 0.056). These results indicate that expression of genes involved in lymphangiogenesis was altered in JSRV-induced lung cancers with *VEGFC* and *VEGFD* under-expressed in cancers as compared to normal lungs.

### Expression of genes from the FGF pathway is unchanged in ovine pulmonary adenocarcinoma except for the overexpression of *FGFR2 mRNA*

**Figure 4:**
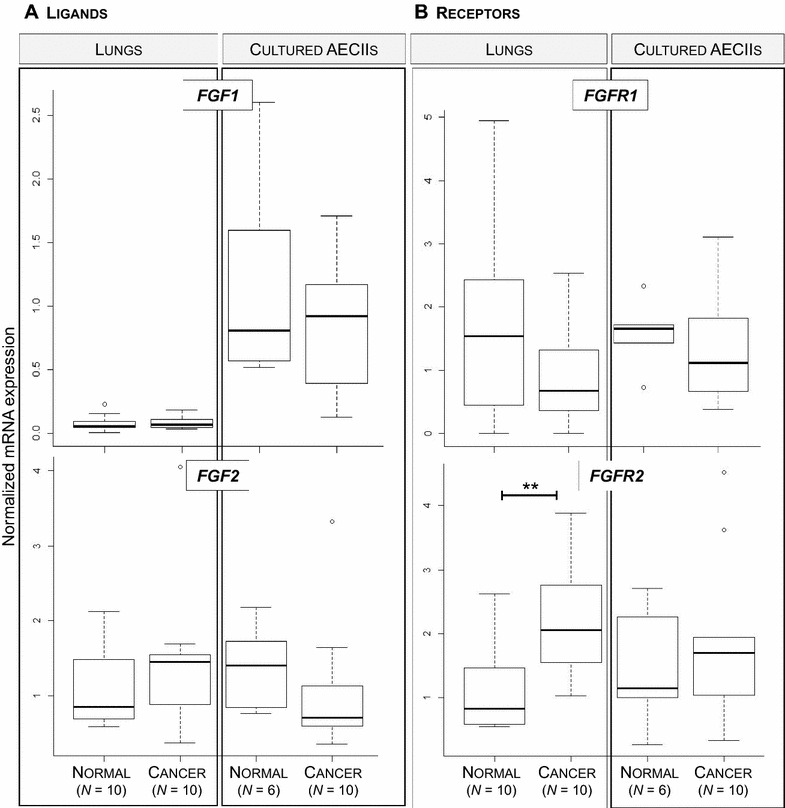
***FGF*** **and** ***FGFR*** **mRNA expression in ovine cancers.** The mRNA expression of *FGF1* and *FGF2* (**A**), *FGFR1* and *FGFR2* (**B**) was quantified by RT-qPCR in lungs and cultured AECII from sheep. Statistical analysis was performed using the Wilcoxon test with **p < 0.005.

We analyzed the expression of genes involved in the FGF pathway by quantification of mRNA expression of the *FGF1* and *FGF2* ligands and of their receptors (*FGFR1* and *FGFR2*) in lungs and primary AECII cultures. No difference of mRNA expression was measured for *FGF1*, *FGF2* and *FGFR1* between normal lungs and cancers (Figure 4)*. FGFR2* mRNA were over-expressed in cancers when compared to normal lungs (Figure 4B). These results highlight that the FGF pathway was unchanged in ovine pulmonary adenocarcinoma except for the over-expression of *FGFR2*.

### The metalloprotease/antiprotease pathway is impaired in JSRV-induced cancers

**Figure 5:**
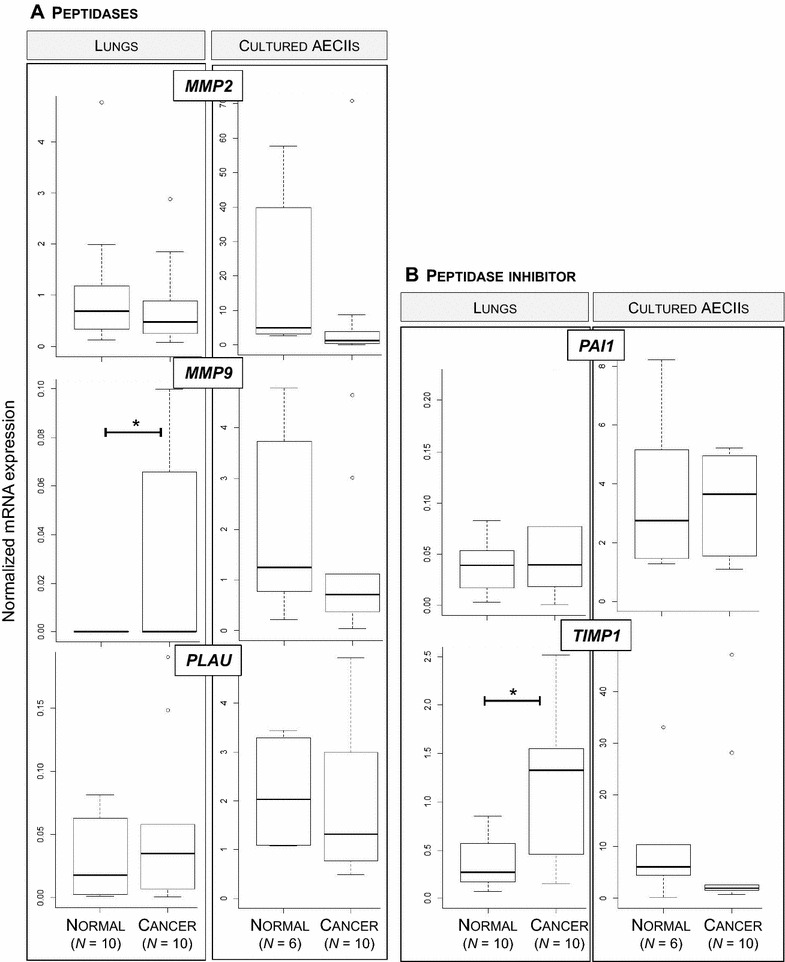
**Expression of genes involved in extracellular matrix degradation in JSRV-induced adenocarcinomas.** Expression of *PLAU*, *MMP2* and *MMP9* (**A**) and *SERPINE1* and *TIMP1* (**B**) mRNA was quantified by RT-qPCR in lungs and lung-derived AECII. Statistical analysis was performed using the Wilcoxon test with *p < 0.05.

Degradation of the extracellular matrix is a major step for cancer extension. Among key genes, mRNA expression of *MMP2*, *MMP9* and *PLAU* peptidases and *TIMP1* and *SERPINE1* peptidase inhibitors has been measured in lungs and cultured AECIIs (Figure 5). No difference in mRNA levels was evidenced in cancers when compared to normal lungs for *MMP2* metalloproteinase, *PLAU* or its inhibitor *SERPINE1* (Figures 5A and B). No expression of *MMP9* was observed in normal samples and only four cancers expressed this gene (Figure 5A). Interestingly, *TIMP1*, one of the metallopeptidase inhibitors, was significantly over-expressed in cancers as compared to normal lungs (Figure 5B). Expression of *MMP2* and *MMP9* metalloproteinases was retained in primary normal AECII (Figure 5A). These results suggest that in JSRV-induced lung adenocarcinomas, mRNA expression of *MMP2* and *MMP9* was reduced in cancer-derived AECII, while not statistically significant, compared to AECII derived from normal lungs. We show that metalloproteinases, that can enhance cell migration by degrading the extracellular matrix, were not overexpressed in cancer except for *MMP9* and that the expression of *TIMP1*, one of their inhibitors, was increased suggesting a blockade of this pathway leading to the degradation of the extracellular matrix.

**Figure 6:**
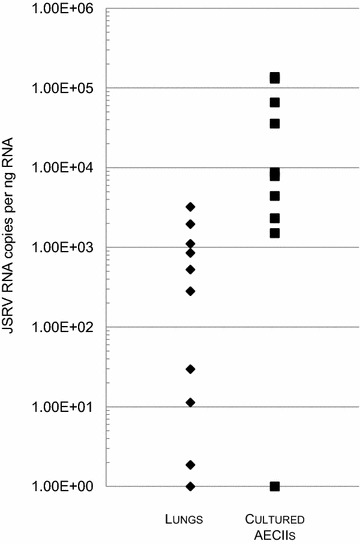
**Quantification of JSRV viral genome in cancers and cancer-derived AECIIs.** JSRV viral RNA have been quantified by RT-qPCR in cancers (*n* = 10) (filled diamond) and cancer-derived AECII cultures (*n* = 10) (filled square). The level of JSRV RNA was expressed as the number of copies per ng of total cell RNA.

Virus load was quantified by quantitative RT-PCR and expressed as the number of copies per ng of total RNA (Figure 6). There was no significant correlation between the expression level of genes implicated in the angiogenic pathway and the number of JSRV RNA copies in lungs (data not shown). This suggests that the observed deregulation of the cellular genes was part of the tumoral process and not directly linked to the level of JSRV replication.

### Dysregulation of the expression of genes implicated in angiogenesis in human lepidic adenocarcinomas

**Figure 7:**
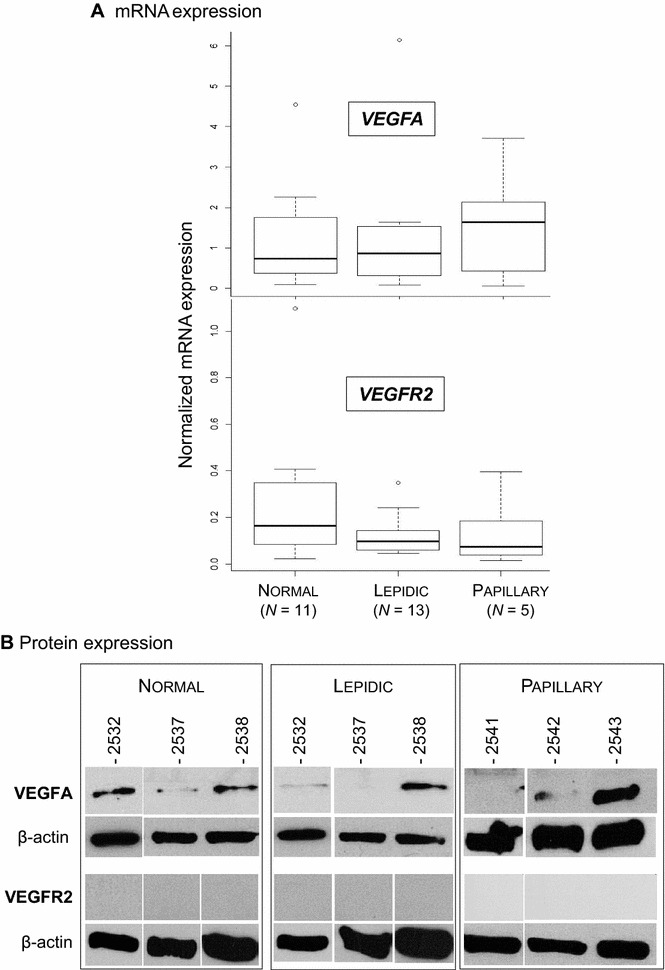
**Lack of** ***VEGFA*** **and** ***VEGFR2*** **mRNA expression in human lepidic and papillary adenocarcinomas. A** mRNA expression of *VEGFA* and *VEGFR2* analyzed by RT-qPCR in normal lungs and lepidic or papillary adenocarcinomas. The mRNA expression of the cellular genes has been normalized with expression of the PPIA, PSMB2 and RPS11 reference genes. **B** VEGFA and VEGFR2 protein expression by Western blot.

We evaluated the levels of expression of key genes in lung tissues collected from lepidic or papillary adenocarcinomas and when available adjacent, non-tumoral lungs from the same patients. Unlike JSRV-induced adenocarcinomas, mRNA (Figure 7A) and proteins (Figure 7B) of *VEGFA* were expressed in human lepidic adenocarcinomas but at very low levels in cancers and in their normal counterparts. VEGFA protein expression was detected in 6/13 (~46%) lepidic adenocarcinomas, 3/5 (60%) papillary adenocarcinoma and 9/11 (~81%) normal lungs. We did not detect differences in the levels of VEGFA mRNA or proteins between lepidic and papillary adenocarcinomas (Figure 7).

Interestingly, we detected a weak (not significant) expression of *VEGFR2*, the main *VEGFA* receptor, in lepidic and papillary tumors (Figure 7). No VEGFR2 protein was detected in lepidic, papillary and normal lung samples (Figure 7). The low mRNA expression of *VEGFA* and the absence of expression of *VEGFR2* in lepidic adenocarcinomas suggest the lack of activation of the angiogenic pathway.

**Figure 8:**
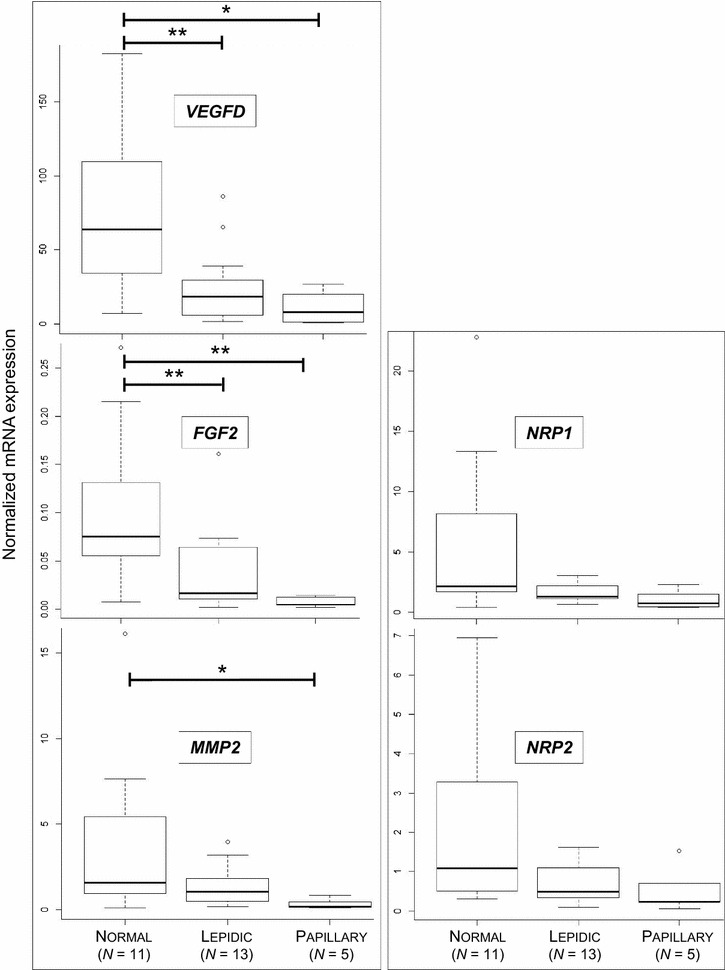
**Expression of other genes involved in angiogenesis in human lepidic and papillary adenocarcinomas.** Expression of human *VEGFD*, *FGF2*, *MMP2*, *NRP1* and *NRP2* mRNA was analyzed by RT-qPCR in normal lungs and lepidic or papillary lung adenocarcinomas. Statistical analysis was performed using Wilcoxon test with *p < 0.05 and **p < 0.005.

The expression of *VEGFD* and *FGF2* mRNA was significantly downregulated in lepidic and papillary adenocarcinomas when compared to adjacent normal lung tissues (Figure 8). Interestingly, mRNA expression of the *MMP2*, *NRP1* and *NRP2* genes was lower (but not significant) in lepidic-type adenocarcinomas as compared to normal tissues from the same patients (Figure 8). Similarly, *MMP2* mRNA expression was significantly lower in papillary-type cancers as compared to normal lungs (Figure 8). Every other gene analyzed in JSRV induced adenocarcinoma was analyzed in human adenocarcinomas; their expression was not different in cancers and normal lungs (data not shown).

Taking all together, this study shows the absence of significant over-expression of genes implicated in metastasis and/or degradation of the extracellular matrix. On the contrary, we observed decreased mRNA expression of *VEGFD*, *FGF2* and *MMP2* and the absence of VEGFR2 proteins in human lepidic adenocarcinomas.

## Discussion

Lung cancer is not a unique disease and can be divided according to different pathological types and subtypes [25]; they may evolve differently in patients and this does influence prognosis and treatment. Most animal models of lung cancers are developed in small rodents. Our work is focused on ovine pulmonary adenocarcinoma, a virally-induced lung cancer in large animals, as a model of human lepidic adenocarcinomas with pneumonic presentation. Both cancers share clinical, radiological and pathological similarities [15]. Predominantly lepidic pulmonary adenocarcinomas are characterized by their development into the lung parenchyma without evidence of stromal, vascular or pleural invasion [15]. Recently, a transgenic mouse model expressing JSRV-Env was developed and exhibited spontaneous lung adenocarcinomas with a metastatic phenotype [26]. These lung cancers were associated with epithelial-mesenchymal transition gene expression upregulation. Our work focused on lepidic-predominant lung adenocarcinomas in sheep and humans, two cancers for which metastatic spread is rare [15], suggesting that the mouse model may not perfectly reproduce the naturally-induced disease.

Cancers need nutrients, oxygen and a way to evacuate metabolic waste to survive just like normal tissues. These functions rely on blood and lymphatic vessels. In the peculiar context of a limited extra-thoracic extension of the cancer cells in predominantly lepidic-type adenocarcinomas, we analyzed the expression of genes involved in angiogenesis, lymphangiogenesis and degradation of the extracellular matrix to decipher this specific feature and we conducted a comparative study in human and ovine lung cancers. The analysis of the VEGFA/VEGFR2 pathway of the main angiogenesis pathway gave some leads to understanding these features. We compared the mRNA expression of the genes of interest in lepidic and in papillary subtypes of human lung cancers, and when available in adjacent normal lungs from lepidic-type adenocarcinomas. For the ovine virally-induced adenocarcinomas, we compared tissues from tumors with positive detection of JSRV genome and JSRV-negative tissues without lesions. When available, we measured the mRNA expression in primary alveolar epithelial type II cells, derived from tumoral or normal tissues as previously described [22]. We identified modulations of the mRNA and protein levels of key genes that might participate in the limitation of extra thoracic metastatic extension in lepidic adenocarcinoma.

We report the absence of VEGFA proteins in JSRV-induced adenocarcinomas in sheep, while the mRNA level of VEGFA was maintained and not different in normal lungs and cancers. *VEGFA* is a key gene during the development of vessels during organogenesis or cancer. Its expression is highly regulated at the translational level [27]. In humans, it has been reported that miRNA regulate *VEGFA* expression. miRNA, such as miRNA-297, miRNA-299, miRNA-567 and miRNA-609 are able to control its expression by preventing protein production without inhibition of the transcription [28]. The existence and activity of this VEGFA-specific miRNA must be explored in sheep, but our data might suggest a similar control in small ruminants. Interestingly, VEGFA protein expression was restored in primary cultures of AECIIs derived from both normal lungs and cancers, proving that these cells retained their potential to produce VEGFA proteins. Our observation may be simply due to the in vitro conditions used to culture and maintain the cells, conditions that do not strictly mimic the in vivo cancer environment. The miRNA activity may also be only efficient in the cancer context since the microenvironment has been reported as an important factor for miRNA inhibition of VEGFA in pancreatic or human colon cancers [29].

We analyzed the mRNA expression of *VEGFR2*, a key receptor for angiogenesis. Interestingly, we observed a significant reduction of *VEGFR2* mRNA and protein expression in ovine lung cancers as compared to normal lungs suggesting a negative control of the receptor in JSRV-induced cancers. The VEGFR2 receptor is mainly present at the surface of the endothelial cells and logically, primary AECII did not express the proteins while the level of mRNA was low to undetectable. Taken together, without VEGFA and VEGFR2, angiogenesis is not activated [4]. Comparatively, we did not see differences in VEGFA expression in human lepidic adenocarcinomas. The VEGFR2 protein was absent in lepidic as well as in papillary adenocarcinomas, while the levels of mRNA stayed very low. To summarize, we show that the main angiogenesis pathway was blocked in ovine JSRV-induced tumors with the absence of VEGFA and VEGFR2 proteins. In human cancers, VEGFA expression was not altered but VEGFR2 was not expressed. Blocking VEGFA and VEGFR2 might contribute to the limitation of extra thoracic dissemination of lepidic-predominant adenocarcinomas in humans and animals. These two proteins, essential for angiogenesis, are targeted by anti-metastasis drugs in various cancers including lung cancers [3].

Alternative pathways play a role in angiogenesis. We then analyzed the expression of other genes implicated in angiogenesis, lymphangiogenesis and degradation of the extracellular matrix. Overall, we measured a reduced expression of *VEGFB* mRNA in cancer-derived AECIIs when compared to cells derived from normal lungs. The expression in tissues was inconstant and low in all cases. Only half of tissues had detectable *VEGFB* mRNA expression. VEGFB is a peculiar angiogenesis ligand. It interacts with VEGFR1 but this interaction does not necessarily activate angiogenesis; the effects of VEGFB vary under specific conditions from induction of endothelial cell permeability to anti-growth and anti-angiogenic effects [30]. Interestingly, the level of *VEGFD* mRNA was lower in cancers than in normal lungs both in human and ovine adenocarcinomas. A significant mRNA down-expression of *VEGFC* and *VEGFD* has been reported in human lung adenocarcinomas [31]. In both tumors, the lower expression of *VEGFD* and the stable expression of *VEGFR3* in tumoral tissues when compared to normal tissues might contribute to the control of metastatic extension.

Aside from the neo-vascularization, tumoral extension necessitates the degradation of the extracellular matrix by cellular peptidases. We measured mRNA expression of peptidases such as *MMP2* and *MMP9*, *PLAU* and of their inhibitors, *SERPINE1* and *TIMP1*. Less than 50% of the JSRV-induced tumors expressed *MMP9* mRNA, otherwise absent in normal tissues, suggesting a blocking of this protease. But interestingly *TIMP1*, an MMP inhibitor, was strongly expressed in tumors. Besides its inhibitory properties, TIMP1 can block the response of endothelial cells to angiogenic factors and participate in the inhibition of endothelial cell migration [32]. We hypothesize that even if some tumors express *MMP9*, the expression of *TIMP1* should block their protease function.

The FGF pathway participates in angiogenesis through its action on cancer cell proliferation [33]. Our results showed perturbation of the mRNA expression of *FGF2* and *FGFR2*. The *FGFR2* expression was significantly higher in tumoral lungs than in normal tissues in sheep and the *FGF2* ligand is down-regulated in human lepidic tumors. Aside from its role in angiogenesis, *FGFR2* has a role in cell proliferation [33]. Our results show that key genes implicated in angiogenesis were impaired during sheep lung tumors and that comparable results were obtained when analyzing this pathway in human lepidic adenocarcinoma.

The ovine pulmonary adenocarcinoma is induced by a retrovirus while the etiology of human lepidic adenocarcinoma is still unknown although a viral etiology has been questioned for decades [16]. Some oncogenic viruses have the ability to directly activate angiogenesis [34] such as EBV (Epstein Barr Virus), HPV (Human Papilloma Virus) and HBV (Hepatitis B Virus) that carry viral proteins with proangiogenic activities [35–37]. To explore a potential direct effect of JSRV proteins on the mRNA levels of the studied genes, we quantified JSRV RNA copies in lungs and AECII cultures. We found no correlation between the level of JSRV expression and the observed deregulation of genes involved in the angiogenesis pathway.

We performed similar analyses in lepidic adenocarcinoma and papillary adenocarcinomas. The results were comparable: VEGFA and VEGFR2 patterns of expression were similar; *VEGFD*, *FGF2* were significantly downregulated as in lepidic adenocarcinomas and *MMP2* was also significantly downregulated. This may be explained by the non-invasive pattern of the cancers analyzed in our study, as shown by their TNM staging.

Taken together, our results indicate that angiogenesis does not represent a hallmark of human and ovine pulmonary adenocarcinomas with predominant lepidic growth. In humans, lepidic adenocarcinoma is a rare lung cancer with no extra thoracic extension. We show that the absence of VEGFA/VEGR2 activation could presumably contribute to the control of extra-thoracic spreading of cancer cells in the virally induced cancer in sheep and in lepidic adenocarcinomas in humans.

The reported benefit of anti-angiogenic agents for the treatment of lung adenocarcinomas at large, may not be observed for patients with lepidic adenocarcinomas or at least for those with no detectable expression of VEGFA and VEGFR2; patients may then avoid potential toxicities that may occur with those agents, that would not be delivered if limited benefit is expected. Ultimately, as we show, JSRV-induced lung adenocarcinoma which naturally occurs in sheep provides a unique model to help to decipher the specific features of lepidic- predominant human lung adenocarcinomas.

## Additional file

**Additional file 1.** **Primers used for detecting mRNAs by quantitative RT-PCR in human and sheep lung adenocarcinomas.** PCR primers for mRNA quantification of ligands, ligand receptor and reference genes have been designed for human and ovine. X: species specificity. Reference sequences are designated by their GenBank #.
