# Supplementary material for: Blocked expression of key genes of the angiogenic pathway in JSRV-induced pulmonary adenocarcinomas
Source: Vet Res. 2017 Nov 14;48:76. doi: 10.1186/s13567-017-0480-z (PMC5686813; doi:10.1186/s13567-017-0480-z)
Supplement: Supplementary file 1 [file 13567_2017_480_MOESM1_ESM.docx]

	Gene	Name	GenBank #	Species: Sheep	Species: Human	5'-3' Forward	5'-3' Reverse
LIGANDS	***VEGFA***	vascular endothelial growth factor A	NM_001025110.1	x		GGGCTGCTGTAATGACGAAAG	TCATCTCTCCTATGTGCTGGC
			NM_001171623.1		x	GCAGAATCATCACGAAGTGGT	GGCACACAGGATGGCTTGA
	VEGFB	vascular endothelial growth factor B	XM_004019872.1	x		CCAGAAGAAAGTGGTGTCATGG	CACTCCAGGCCATCGTCAG
			NM_003377.4		x	CTGACGATGGCCTGGAGTGT	AGGTCTGCATTCACACTGGC
	VEGFC	vascular endothelial growth factor C	XM_004021836.1	x	x	ATGTGGGGAAGGAGTTTGGAG	CTGGTTTGGGGCCTTGAGAG
	VEGFD	vascular endothelial growth factor D	XM_004021929.1	x		GAGCCTCGTCTGTGTCAATAC	GATCTTCTTCTGGGATCTGGATGG
			NM_004469.4		x	GGAGACCTGCTGCCAGAAGCAC	GCCCCTGGGCAGCCCTTTT
	FGF1	fibroblast growth factor 1	XM_004008910.1	x		GTGGATGGGACGAAGGACAG	CTGGGTGTTTGTGAGCCGTA
			NM_000800.4		x	GGAGCGACCAGCACATTCAG	GGTTCTCCTCCAGCCTTTCC
	FGF2	fibroblast growth factor 2	NM_001009769	x		GCAGAAGAGAGAGGGGTTGT	CCCAGTTCGTTTCAGTGCCA
			NM_002006.4		x	GCAGAAGAGAGAGGAGTTGT	CCCAGTTCGTTTCAGTGCCA
RECEPTORS	***VEGFR1***	vascular endothelial growth factor receptor 1	XM_004012285.1	x		TTCCCTGCTTGATGACTACCA	AAACGTGGGTCTGCTGAGG
			NM_002019.4		x	ACATACTCAACTCCTGCCTTCTC	TCGCCCTGGTAGTCATCAAAC
	VEGFR2	vascular endothelial growth factor receptor 2	NM_001278565	x		AAGACAGCAACCTGCAAGAC	GGTACAGGCACCAAGAAGATTG
			NM_002253.2		x	TCCCAGATGACAACCAGACG	GCCTTCAGATGCCACAGACT
	VEGFR3	vascular endothelial growth factor receptor 3	XM_012177600.1	x	x	CCGAGGGATGGAGTTCCTG	AGGGTCCTTGTAGATGTCCC
	FGFR1	fibroblast growth factor receptor 1	XM_012123734.1	x		GGATCTACACCCACCAGAGC	GCTTGTCCATACGATGACCCT
			NM_023110.2		x	GCTGACTCCAGTGCATCCAT	GGGGTTTGCCTAAGACCAGT
	FGFR2	fibroblast growth factor receptor 2	XM_012103034.1	x		GTGATGTCTGGTCCTTCGGG	GGTCTCTGTGAGGGTACTGC
			NM_000141.4		x	CCTGGATGTTGTGGAGCGAT	GCCCGTATTTACTGCCGTTC
	NRP1	neuropilin 1	XM_004014210.1	x	x	TCAGGATCACACAGGAGATGG	GTCATGCAGTGGGCAGAGTT
	NRP2	neuropilin 2	XM_004004851.1	x		TGCACTATGACACCCCTGAC	TGTGGGCTTTGAGTCTGTCC
			XM_005246934.2		x	TGCACTATGACACCCCTGAC	CGTGGGCTTGGAGTCTGTCC
PEPTIDASES/ PEPTIDASE INHIBITORS	***MMP2***	matrix metallopeptidase 2	NM_001166180.1	x		GGCATCTCTCAGATCCGTGG	CCTTCTCCTCCTGTGGGTCT
			NM_004530.5		x	TCATTTGGCGGACTGTGACG	AGGGTGCTGGCTGAGTAGAT
	MMP9	matrix metallopeptidase 9	XM_004014614	x		GCGCTGGGCTTAGATCACTC	TCAGGGCGAGGACCATACA
			NM_004994.2		x	TTCCAGTACCGAGAGAAAGCCTAT	GGTCACGTAGCCCACTTGGT
	PLAU	plasminogen activator, urokinase	NM_001163593.1	x		GGTGTATCGGACTGTGGCTG	TGAGGTCTGTGTTGGCCTTC
			NM_002658.3		x	CTGCCTGCCCTCGATGTATAAC	CTTCAGAGCCGTAGTAGTGGG
	TIMP1	metallopeptidase inhibitor 1	NM_001009319.2	x		TACTTCCACAGGTCCCAGAAT	GCAGGGGATAGATGAGCAGG
			NM_003254.2		x	TACTTCCACAGGTCCCACAAC	GCAGGGGATGGATAAACAGG
	SERPINE1	serpin family E member 1	NM_001174114	x		CCCCATGAGAAAGAGGTGCC	AGGGAGAACTTGGGCAGAAC
			NM_000602.4		x	TGCAGAAAGTGAAGATCGAGGT	CCAGGGTCAGGGTTCCATCA
REFERENCE	***PPIA***	peptidyl isomerase A	XM_004013990	x		GTGCCAGGGTGGTGACTTC	CCACATGCTTGCCATCCAAC
			NM_001300981.1		x	GTGTCAGGGTGGTGACTTC	CCACATGCTTGCCATCCAAC
	PSMB2	proteasome subunit beta 2	XM_004001790.1	x		GCTATGATGAGCACGAGGGT	CGCGAGATAGTTGGCGTGTAA
			NM_002794		x	TCACGTGAGAGGGCAGTGGAAC	CCCTGGCAAGTGGGAGGGAG
	RPS11	ribosomal protein S11	NM_001015.4		x	TGCACTACATCCGCAAGTACA	CACGTTGAAGCGCACTGTCT
	YWHAZ	tyrosine 3-monooxygenase/tryptophan 5-monooxygenase activation protein, zeta	NC_019466	x		AACTCCCCTGAGAAAGCCTG	CCGATGTCCACAATGTCAAGTT
